# The impact of KRAS mutations on prognosis in surgically resected colorectal cancer patients with liver and lung metastases: a retrospective analysis

**DOI:** 10.1186/s12885-016-2141-4

**Published:** 2016-02-18

**Authors:** Hae Su Kim, Jin Seok Heo, Jeeyun Lee, Ji Yun Lee, Min-Young Lee, Sung Hee Lim, Woo Yong Lee, Seok Hyung Kim, Yoon Ah Park, Yong Beom Cho, Seong Hyeon Yun, Seung Tae Kim, Joon Oh Park, Ho Yeong Lim, Yong Soo Choi, Woo Il Kwon, Hee Cheol Kim, Young Suk Park

**Affiliations:** Division of Hematology-Oncology, Department of Medicine, Samsung Medical Center, Sungkyunkwan University School of Medicine, Seoul, Korea; Surgery, Samsung Medical Center, Sungkyunkwan University School of Medicine, Seoul, Korea; Pathology, Samsung Medical Center, Sungkyunkwan University School of Medicine, Seoul, Korea; Thoracic Surgery, Samsung Medical Center, Sungkyunkwan University School of Medicine, Seoul, Korea; Division of Hematology-Oncology, Department of Medicine, Veterans Health Service Medical Center, Seoul, Korea

**Keywords:** Colorectal cancer, KRAS mutation, Prognosis, Metastases

## Abstract

**Background:**

*KRAS* mutations are common in colorectal cancer (CRC). The role of *KRAS* mutation status as a prognostic factor remains controversial, and most large population-based cohorts usually consist of patients with non-metastatic CRC. We evaluated the impact of *KRAS* mutations on the time to recurrence (TTR) and overall survival (OS) in patients with metastatic CRC who underwent curative surgery with perioperative chemotherapy.

**Methods:**

Patients who underwent curative resection for primary and synchronous metastases were retrospectively collected in a single institution during a 6 year period between January 2008 and June 2014. Patients with positive surgical margins, those with known *BRAF* mutation, or those with an unknown *KRAS* mutation status were excluded, and a total of 82 cases were identified. The pathological and clinical features were evaluated. Patients’ outcome with *KRAS* mutation status for TTR and OS were investigated by univariate and multivariate analysis.

**Results:**

*KRAS* mutations were identified in 37.8 % of the patients and not associated with TTR or OS between *KRAS* wild type and *KRAS* mutation cohorts (log-rank *p* = 0.425 for TTR; log-rank *p* = 0.137 for OS). When patients were further subdivided into three groups according to mutation subtype (wild-type *vs. KRAS* codon 12 mutation *vs. KRAS* codon 13 mutation) or amino acid missense mutation type (G > A *vs*. G > T *vs*. G > C), there were no significant differences in TTR or OS. Mutational frequencies were significantly higher in patients with lung metastases compared with those with liver and ovary/bladder metastases (*p* = 0.039), however, *KRAS* mutation status was not associated with an increased risk of relapsed in the lung.

**Conclusions:**

*KRAS* mutation was not associated with TTR or OS in patients with metastatic CRC who underwent curative surgery with perioperative chemotherapy.

## Background

Colorectal cancer (CRC) is the fourth leading cause of cancer-related death worldwide [1]. Although the development of molecular-targeted therapy has improved the survival of patients with metastatic CRC [2, 3], the majority of patients with stage IV CRC who undergo complete resection die from metastatic disease. Nevertheless, a good proportion of patients demonstrate good recurrence-free survival. CRC tumorigenesis is characterized by the accumulation of genetic alterations, and V-Ki-ras2 Kirsten rat sarcoma viral oncogene homolog (*KRAS*) mutations are an early event in tumorigenesis [4]. *KRAS* mutations occur in approximately 30 to 40 % of patients with CRC, and 90 % of *KRAS* mutations occur in codon 12 or 13 [2, 5, 6]. *KRAS* mutations lead to constitutive activation of downstream pathways, including the Ras/Raf/MAP/MEK/ERK and/or PTEN/PI3K/Akt pathways [7–10]. *KRAS* mutations are established biomarkers for predicting the poor efficacy of anti-epidermal growth factor receptor (*EGFR*) monoclonal antibodies in patients with stage IV CRC [2, 5, 11], but the prognostic relevance of *KRAS* mutations remains controversial [12–16]. Recent studies, in patients with resected stage II and/or III CRC, have highlighted the prognostic value of *KRAS* codon12 and 13 mutations, showing correlations between mutation subtype, cancer recurrence, and poor overall survival [13–15].

Large population-based cohorts usually consist of patients with non-metastatic CRC [12, 14, 16, 17]. The prognostic impact of *KRAS* mutation in patients with synchronous metastatic CRC who undergo curative resection with perioperative chemotherapy is unknown. The current study investigated the impact of *KRAS* mutations on the time to recurrence (TTR) and overall survival (OS) in patients with stage IV CRC who underwent curative surgery with perioperative chemotherapy. In addition, the recurrence pattern according to *KRAS* mutation status after complete resection was evaluated.

## Methods

### Patients

In this retrospective study, patients who underwent curative resection for primary and synchronous metastases at our institution between January 2008 and June 2014 were identified from the hospital records. Patients who underwent separate colorectal resection and metastasectomy were excluded if the duration between the two procedures exceeded 2 months. Patients with positive surgical margins, those with known v-Raf murine sarcoma viral oncogene homolog B (*BRAF*) mutations, or those with an unknown *KRAS* mutation status were also excluded. All patients included in the study were administered 5-FU with/without oxaliplatin or irinotecan-based chemotherapy. Clinical and pathological data including sex, patient age, tumor location, resection site, staging at surgery (performed in accordance with the classification of the 6th Edition of the American Joint Committee on Cancer guidelines), *BRAF* mutation status, perioperative chemotherapy regimens, use of molecular targeting agents including cetuximab and bevacizumab, were collected. The study protocol was reviewed and approved by the SMC institutional review board.

### Perioperative chemotherapy regimens

Oxaliplatin based chemotherapy was FOLFOX (oxaliplatin 85 mg/m^2^ on day 1, infused during 2 h; LV 200 mg/m^2^, infused during 2 h, followed by 5-FU as a 400 mg/m^2^ intravenous bolus then a 1200 mg/m^2^ infusion during 22 h on days 1 and 2) in 2 week treatment cycles or XELOX(oxaliplatin 130 mg/m^2^ on day 1 followed by oral capecitabine 1000 mg/m^2^ twice daily (day 1 to 14) in 3 week treatment cycles. Irinotecan based chemotherapy was FORFIRI (irinotecan 180 mg/m^2^ on day 1, infused during 2 h; LV 200 mg/m^2^, infused during 2 h, followed by 5-FU as a 400 mg/m^2^ intravenous bolus then a 1200 mg/m^2^ infusion during 22 h on days 1 and 2) in 2 week treatment cycles or XELIRI (irinotecan 250 mg/m^2^ on day 1 followed by oral capecitabine 1000 mg/m^2^ twice daily (day 1 to 14) in 3 week treatment cycles. If bevacizumab or cetuximab was used, patients received cetuximab (initial dose 400 mg/m^2^ infused during 2 h, and 250 mg/m^2^ weekly) or bevacizumab (5 mg/kg) followed by FOLFOX or FOLFIRI.

### DNA extraction and mutation analysis

DNA was isolated from 10-μm formalin-fixed, paraffin-embedded tumor specimens using FFPE-DNA isolation kit (Qiagen, Hilden, Germany). A Qiagen the rascreen *KRAS* mutation kit was used to detect the seven most common *KRAS* codon 12 and 13 mutations. Specifically, the mutation was detected by real-time polymerase chain reaction based on amplification-refractory mutation system and Scorpion probes (Gly12Asp [GGT > GAT] G12D, Gly12Val [GGT > GAC] G12V, Gly12Cys [GGT > TGT] G12C, Gly12Ser [GGT > AGT] G12S, Gly12Ala [GGT > GCT] G12A, Gly12Arg [GGT > CGT] G12R, Gly13Asp [GGC > GAC] G13D).

### Statistical analyses

Patients were subdivided into wild-type *KRAS* and mutant *KRAS* cohorts. The primary objective was to investigate the effect of *KRAS* mutation on the TTR. TTR was defined as the time from the date of operation to the date of local or metastatic recurrence. As of November 2014, overall survival data are not yet available for the mutant KRAS group. Data from recurrence-free patients were censored at the date of the last follow-up.

To compare baseline characteristics, categorical outcomes were analyzed using the chi-square test or Fisher’s exact test. Continuous variables are presented as medians and ranges. TTR and OS were calculated using the Kaplan-Meier method, and data was compared using the log-rank test. The Cox proportional hazard model was used to assess hazard ratios (HRs) of prognostic factor. All factors of statistical significance (*p* < 0.10) in univariate analysis were included in the multivariate analysis. Two-sided *p* values of <0.05 were considered as statistically significant. All statistical analyses were performed using the SPSS statistical software version 21 (IBM, Armonk, NY. USA).

## Results

### Patient characteristics

Between January 2008 and June 2014, 82 patients who were diagnosed with synchronous metastatic CRC and underwent curative resection of primary and metastatic lesions with perioperative chemotherapy were included in the analyses. Table 1 summarizes the patient characteristics according to *KRAS* mutation status. There was no significant difference in clinicopathologic features between the two groups. Baseline characteristics including age, sex, tumor location, tumor grade, T stage, N stage, synchronous metastasectomy site, and recurrence site were similar between the *KRAS* wild type and *KRAS* mutation cohorts. Regarding BRAF mutation status, all of the tested cases (76.8 %) were *BRAF* wild type.

**Table 1:** Baseline characteristics according to *KRAS* mutation status

Characteristics	No. of patients	*KRAS*		
		wild-type	mutant	*p*-value
	(*n* = 82)	(*n* = 51)	(*n* = 31)	
Age, year, Median (range)	55.8 (25–77)	58.8 (25–77)	55.5 (29–77)	0.565
≥65 years	17 (21 %)	12 (24 %)	5 (16 %)	0.423
Sex				0.867
Male	44 (54 %)	27 (53 %)	17 (55 %)	
Female	38 (46 %)	24 (47 %)	14 (45 %)	
Location				0.246
Colon	54 (66 %)	36 (71 %)	18 (58 %)	
Rectum	28 (34 %)	15 (29 %)	13 (42 %)	
Neoadjuvant Chemotherapy	21 (26 %)	11 (22 %)	10 (32 %)	0.282
Resection site				0.039
Liver	57 (69 %)	39 (76 %)	18 (58 %)	
Lung	13 (16 %)	4 (8 %)	9 (29 %)	
Others (ovary, bladder)	12 (15 %)	8 (16 %)	4 (13 %)	
Tumor grade				0.432
Well	10 (12 %)	7 (14 %)	3 (10 %)	
Moderate/Poor	72 (78 %)	44 (86 %)	28 (90 %)	
T stage				0.265
T1	1 (1 %)	1 (2 %)	0 (0 %)	
T2	2 (2 %)	2 (4 %)	0 (0 %)	
T3	47 (57 %)	30 (59 %)	17 (55 %)	
T4	30 (37 %)	18 (35 %)	12 (39 %)	
Tx	2 (2 %)	0 (0 %)	2 (6 %)	
N stage				0.824
N0	12 (15 %)	8 (16 %)	4 (13 %)	
N1	31 (38 %)	18 (35 %)	13 (42 %)	
N2	39 (47 %)	25 (49 %)	14 (45 %)	
1st Adjuvant Chemo-Regimen				0.923
Oxaliplatin-based	70 (86 %)	44 (86 %)	26 (84 %)	
Irinotecan-based	10 (12 %)	6 (12 %)	4 (13 %)	
Only 5-FU	2 (2 %)	1 (2 %)	1 (3 %)	
Use of Cetuximab at 1st post-operative chemotherapy	4 (5 %)	4 (8 %)	0 (0 %)	NA
Use of Becavizumab at 1st post-operativechemotherapy	13 (16 %)	6 (12 %)	7 (23 %)	0.194
Ever use of Cetuximab	16 (20 %)	16 (31 %)	0 (0 %)	NA
Ever use of Bevacizumab	23 (28 %)	10 (20 %)	13 (42 %)	0.029
Recurrence pattern (*n* = 57)				0.616
Primary site	3 (5 %)	1 (2 %)	2 (8 %)	
Metastasectomy site	27 (47 %)	15 (46 %)	12 (50 %)	
New distant sites	27 (47 %)	17 (52 %)	10 (42 %)	
Duration of follow up month, median (range)	25 (4–74)	25 (4–74)	34 (9–63)	0.763

*Abbreviations*: *CI* confidence interval, *A.A* amino acid

### Subtype of *KRAS* mutations

Of 82 patients, *KRAS* mutations were detected in 31 (37.8 %) patients. Eighteen (58 %) patients harbored codon 12 mutations including 9 with c.35G > A (p.G12D, codon 12 GGT > GAT), 5 with c.35G > T (pG12V, codon 12 GGT > GTT), 2 with c.35G > C (p.G12A, codon 12 GGT > GCT), and 2 with c.34G > A (p.G12S, codon 12 GGT > AGT). For the 13 (42 %) patients with codon 13 mutations, all had the c.38G > A (p.G13D, codon 13 GGC > GAC) mutation. *KRAS* amino acid mutations were also analyzed. The G > A missense mutation was the most frequently observed mutation, followed by the G > T and G > C mutations.

### The impact of *KRAS* mutations on TTR and OS

**Fig. 1:**
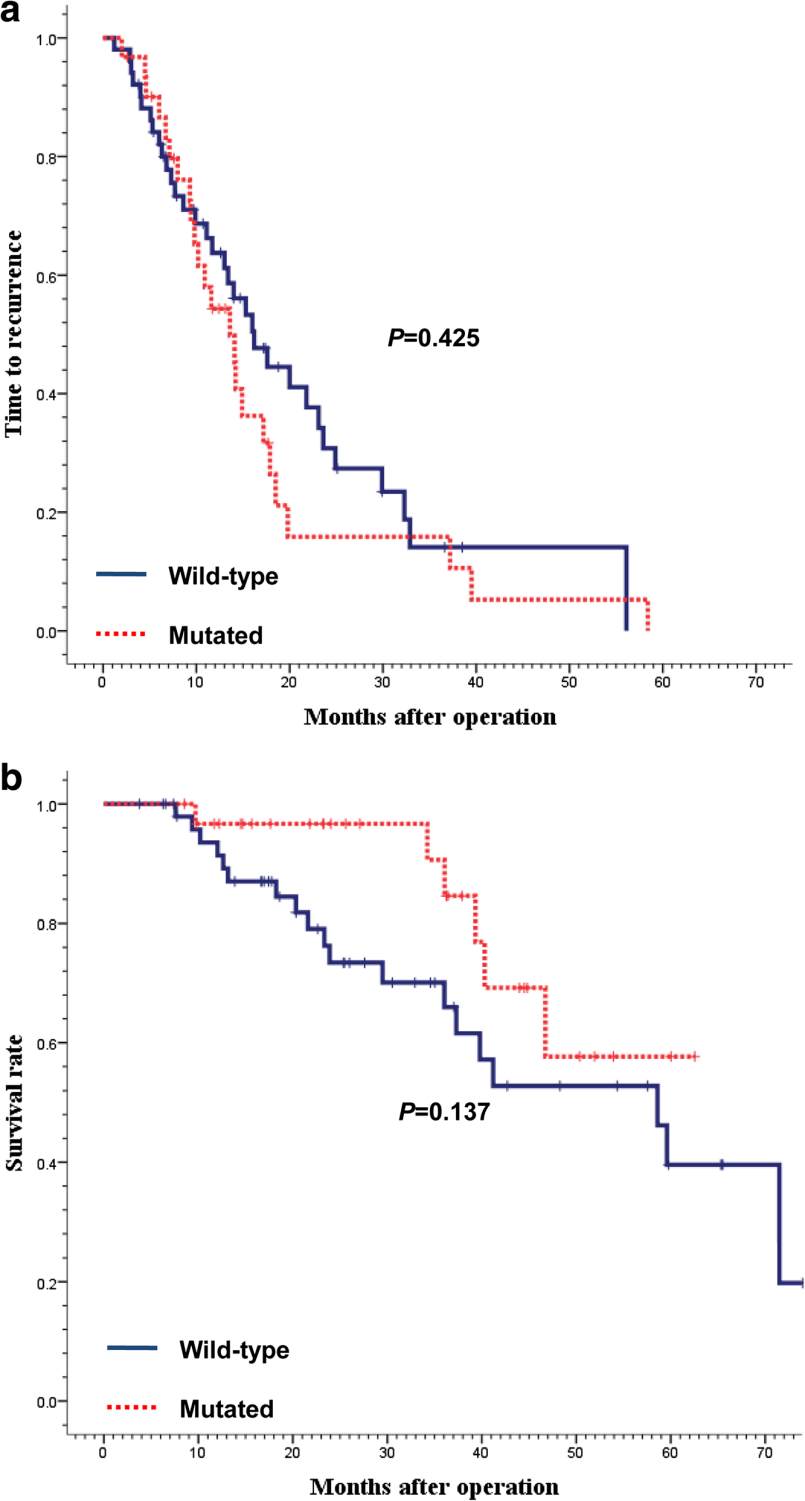
Time to recurrence (**a**) and overall survival (**b**) according to *KRAS* status. KRAS mutation status had no impact on time to recurrence (*p* = 0.425) and overall survival (*p* = 0.137)

The median follow-up durations were 25 months (range, 4–74) and 34 months (range, 9–63) for patients with *KRAS* wild type and *KRAS* mutation status, respectively. During follow-up in surviving participants, there were 57 events for TTR analysis and 25 events for OS analysis. There were no significant differences in survival time distributions according to *KRAS* wild type and *KRAS* mutation status (log-rank *p* = 0.425 for TTR; log-rank *p* = 0.137 for OS, Fig. 1). In univariate and multivariate analyses, there were no significant differences in TTR or OS between *KRAS* wild type and *KRAS* mutation cohorts (Tables 2, 3 and 4). When patients were further subdivided into three groups according to mutation subtype (wild-type *vs. KRAS* codon 12 mutation *vs. KRAS* codon 13 mutation) or amino acid missense mutation type (G > A vs. G > T vs. G > C), there were no significant differences in TTR or OS.

**Table 2:** Univariate analysis for time to recurrence

Characteristics	Hazard ratio (95 % CI)	*p*-value
Location of primary tumor (rectum *vs* colon)	0.956 (0.548–1.669)	0.875
Age (≥65 *vs* <65)	0.856 (0.418–1.755)	0.671
Sex (female *vs* male)	0.678 (0.399–1.150)	0.150
Neoadjuvant chemotherapy (Yes *vs* No)	1.040 (0.563–1.923)	0.899
Tumor grade (moderate/poor *vs* well)	1.201 (0.508–2.843)	0.676
T stage (T4 *vs* T1-3)	1.041 (0.608–1.782)	0.885
N stage (N2 *vs* N0,1)	1.197 (0.703–2.037)	0.508
Resection site		
Liver	1	
Lung	0.694 (0.311–1.550)	0.373
Others (ovary, uterus, bladder)	0.670 (0.299–1.502)	0.331
Use of Cetuximab at 1st post-operative chemotherapy (Yes *vs* No)	0.589 (0.143–2.425)	0.463
Use of Bevacizumab at 1st post-operative chemotherapy (Yes *vs* No)	0.582 (0.231–1.469)	0.252
*KRAS* (mutation *vs* wild)	1.245 (0.725–2.137)	1.245
*KRAS* subtype		
Wild	1	
12th	1.127 (0.599–2.123)	0.710
13th	1.230 (0.561–2.697)	0.605
A.A Mutation type		
Wild (*n* = 51)	1	
Guanine to thymidine (*n* = 5)	0.737 (0.164–3.315)	0.691
Guanine to cytosine (*n* = 2)	1.482 (0.766–2.864)	0.242
Guanine to adenine (*n* = 24)	1.029 (0.553–1.931)	0.928

*Abbreviations*: *CI* confidence interval, *A.A* amino acid, *HR* hazard ratio

**Table 3:** Univariate analysis for overall survival

Characteristics	HR (95 % CI)	*p*-value
Location of primary tumor (rectum *vs* colon)	0.531 (0.212–1.333)	0.178
**Age (≥65** *** vs*** ** <65)**	**7.492 (2.941–9.084)**	**<0.001**
**Sex (female** *** vs*** ** male)**	**2.038 (0.908–4.578)**	**0.085**
Neoadjuvant chemotherapy (Yes *vs* No)	1.114 (0.460–2.698)	0.811
Tumor grade (moderate/poor *vs* well)	1.332 (0.312–5.693)	0.698
**T stage (T4** *** vs*** ** T1-3)**	**4.324 (1.857–10.068)**	**0.001**
N stage (N2 *vs* N0,1)	1.906 (0.854–4.251)	0.115
Resection site		
Liver	1	
Lung	0.311 (0.041–2.335)	0.256
Others (ovary, uterus, bladder)	1.036 (0.345–3.108)	0.950
**Use of Cetuximab at 1st post-operative chemotherapy (Yes** *** vs*** ** No)**	**3.777 (0.850–16.779)**	**0.081**
Use of Bevacizumab at 1st post-operative chemotherapy (Yes *vs* No)	0.899 (0.267–3.027)	0.863
*KRAS* (mutation *vs* mutation)	0.500(0.198–1.267)	0.144
*KRAS*		
Wild	1	
12th	0.330 (0.076–1.428)	0.138
13th	0.675 (0.227–2.010)	0.481

*Abbreviations*: *CI* confidence interval, *A.A* amino acid, *HR* hazard ratio

Factors of statistical significance (*p* < 0.10) in univariate analysis presented with boldface

**Table 4:** Multivariate analysis for overall survival

Characteristics	HR (95 % CI)	*p*-value
Age (≥65 *vs* <65)	9.749 (3.404–27.919)	<0.001
Sex (female *vs* male)	3.070 (1.260–7.478)	0.014
T stage (T4 *vs* T1-3)	3.511 (1.484–8.307)	0.004
Use of Cetuximab at 1st post-operative chemotherapy (Yes *vs* No)	1.185 (0.235–5.979)	0.837

*Abbreviations*: *CI* confidence interval *A.A* amino acid; HR, hazard ratio

### The effect of *KRAS* mutation status on the recurrence site

Mutational frequencies were significantly higher in patients with lung metastases compared with those with liver and ovary/bladder metastases (*KRAS* mutant: lung 9/13 [69 %], liver 18/57 [31 %], ovary/bladder 4/12 [33 %]; *p* = 0.039). However, *KRAS* mutation status was not associated with an increased risk of relapse in the lung, and the majority of recurrence occurred at the previous metastasectomy sites (15/33 *vs*. 24/31 for *KRAS* wild type *vs. KRAS* mutation, respectively).

## Discussion

The majority of studies evaluating the prognostic impact of *KRAS* mutational status in CRC have been conducted in patients with stage II/III disease. The QUASAR trial, which mainly evaluated patients with stage II CRC, revealed that *KRAS* mutations had a detrimental effect on recurrence and OS, despite adjuvant chemotherapy [17]. In contrast, the CALGB 89803 and PETACC-3 trials demonstrated that *KRAS* mutation status had no significant effect on recurrence or OS in patients with stage II/III colon cancer or CRC treated with adjuvant chemotherapy [12, 16]. However, conflicting findings were reported simultaneously in two large studies conducted by The Kirsten ras in-colorectal-cancer collaborative group, the RASCAL and RASCAL II trials, which were comprised of 2721 and 4268 patients, respectively [18, 19]. Although the first RASCAL study reported an association of *KRAS* mutations with an increased risk of recurrence and death for patients with all stages of CRC, recurrence in patients with Dukes’ D tumors was less than might be expected. The RASCAL II study concluded that there was a significant prognostic value in failure-free survival alone in patients with Dukes’ C cancer harboring a *KRAS* G12V mutation.

Few studies have evaluated the relationship between patients with stage IV disease at the time of diagnosis and *KRAS* mutations [20–23]. Patients with metastatic CRC with limited metastases undergo curative primary resection with or without metastasectomy, anti-EGFR antibody therapy, and heterogeneous chemotherapy regimens, making it difficult to evaluate the precise prognostic value of *KRAS* status in this setting. To overcome this limitation, in this study, we included only patients who underwent curative resection of the primary and metastatic sites who received perioperative chemotherapy. To our knowledge, this study is the first to report TTR in such patients. In this homogenous cohort of Korean patients with metastatic CRC, we observed that *KRAS* mutation was not associated with TTR or OS, which is congruent with previous studies [20–22]. Phipps et al., reported that *KRAS* mutations did not differ by stage at diagnosis, and that the prognostic value of *KRAS* mutations only became evident in patients with stage I-III disease [22]. Furthermore, Nash et al., reported that the prevalence of *KRAS* mutations did not vary with stage, but that *KRAS* mutations were strong independent predictors of survival for patients with stage I-III CRC [21].

We also investigated the association *KRAS* mutations with recurrence pattern in our cohort. *KRAS* mutations were significantly more common in lung metastases compared with liver and bladder/ovary metastases. These finding were concordant with those of Tie et al., who observed a significantly higher prevalence of *KRAS* mutations in patients with lung metastases compared with those with liver metastases [24]. In addition, in their study, *KRAS* mutations were associated with an increased risk of lung relapse in patients with stage II/III CRC who were enrolled on the VICTOR clinical trial [21]. However, in the present study, we did not observe recurrence-specific associations with *KRAS* mutation status. The differential impact of *KRAS* mutations on recurrence-specific sites according to disease stage requires evaluation in further studies.

Limitations of the present study included the relatively short follow-up, where the median OS was not reached in the *KRAS* mutation group. Nevertheless, sufficient TTR events occured enabling analysis of recurrence. In addition, the *BRAF* mutation status was not determined for 19 (33 %) patients, but *BRAF* mutations were only detected in a small proportion of patient and were not significantly different between *KRAS* wild type and *KRAS* mutated patients. In addition, the small sample size did not allow us to evaluate the impact of different *KRAS* mutation subtypes.

In conclusion, *KRAS* mutation was not associated with TTR or OS in curatively resected, metastatic CRC. Further validation of these finding is needed in metastatic CRC patients treated with curative resection in prospective controlled trials.

## Conclusions

The present study, to our knowledge, is the first report on the effect of *KRAS* mutations on prognosis in surgically treated CRC patients with synchronous metastases. The most of previous studies evaluating the prognostic impact of KRAS in CRC have been conducted in patients with non-metastatic CRC, and the influence of *KRAS* mutations on outcome is conflicting. In our study, *KRAS* mutation was not associated with TTR or OS in metastatic CRC patients who undergo curative surgery and perioperative chemotherapy. *KRAS* mutation status was also not linked to recurrence pattern. Prospective studies will be necessary to evaluate the prognostic effect of *KRAS* mutation in metastatic CRC patients.

### Consent

This research is strictly retrospective and involving the collection of existing data and records. The study protocol was reviewed and approved consent exemptions by the SMC institutional review board.

## Abbreviations

BRAF: v-Raf murine sarcoma viral oncogene homolog B
CIs: Confidence intervals
CRC: Colorectal cancer
EGFR: Epidermal growth factor receptor
KRAS: V-Ki-ras2 Kirsten rat sarcoma viral oncogene homolog
OS: Overall survival
TTR: Time to recurrence

## References

[1] Siegel R, Ward E, Brawley O, Jemal A (2011). Cancer statistics, 2011: the impact of eliminating socioeconomic and racial disparities on premature cancer deaths. CA Cancer J Clin.

[2] Karapetis CS, Khambata-Ford S, Jonker DJ, O’Callaghan CJ, Tu D, Tebbutt NC (2008). K-ras mutations and benefit from cetuximab in advanced colorectal cancer. N Engl J Med.

[3] Hurwitz H, Fehrenbacher L, Novotny W, Cartwright T, Hainsworth J, Heim W (2004). Bevacizumab plus irinotecan, fluorouracil, and leucovorin for metastatic colorectal cancer. N Engl J Med.

[4] Vogelstein B, Fearon ER, Hamilton SR, Kern SE, Preisinger AC, Leppert M (1988). Genetic alterations during colorectal-tumor development. N Engl J Med.

[5] Van Cutsem E, Kohne CH, Hitre E, Zaluski J, Chang Chien CR, Makhson A (2009). Cetuximab and chemotherapy as initial treatment for metastatic colorectal cancer. N Engl J Med.

[6] Nosho K, Irahara N, Shima K, Kure S, Kirkner GJ, Schernhammer ES (2008). Comprehensive biostatistical analysis of CpG island methylator phenotype in colorectal cancer using a large population-based sample. PLoS One.

[7] Bos JL, Fearon ER, Hamilton SR, Verlaan-de Vries M, van Boom JH, van der Eb AJ (1987). Prevalence of ras gene mutations in human colorectal cancers. Nature.

[8] Di Fiore F, Blanchard F, Charbonnier F, Le Pessot F, Lamy A, Galais MP (2007). Clinical relevance of KRAS mutation detection in metastatic colorectal cancer treated by Cetuximab plus chemotherapy. Br J Cancer.

[9] Benvenuti S, Sartore-Bianchi A, Di Nicolantonio F, Zanon C, Moroni M, Veronese S (2007). Oncogenic activation of the RAS/RAF signaling pathway impairs the response of metastatic colorectal cancers to anti-epidermal growth factor receptor antibody therapies. Cancer Res.

[10] Wan PT, Garnett MJ, Roe SM, Lee S, Niculescu-Duvaz D, Good VM (2004). Mechanism of activation of the RAF-ERK signaling pathway by oncogenic mutations of B-RAF. Cell.

[11] Bokemeyer C, Bondarenko I, Makhson A, Hartmann JT, Aparicio J, de Braud F (2009). Fluorouracil, leucovorin, and oxaliplatin with and without cetuximab in the first-line treatment of metastatic colorectal cancer. J Clin Oncol.

[12] Roth AD, Tejpar S, Delorenzi M, Yan P, Fiocca R, Klingbiel D (2010). Prognostic role of KRAS and BRAF in stage II and III resected colon cancer: results of the translational study on the PETACC-3, EORTC 40993, SAKK 60–00 trial. J Clin Oncol.

[13] Imamura Y, Morikawa T, Liao X, Lochhead P, Kuchiba A, Yamauchi M (2012). Specific mutations in KRAS codons 12 and 13, and patient prognosis in 1075 BRAF wild-type colorectal cancers. Clin Cancer Res.

[14] Blons H, Emile JF, Le Malicot K, Julie C, Zaanan A, Tabernero J (2014). Prognostic value of KRAS mutations in stage III colon cancer: post hoc analysis of the PETACC8 phase III trial dataset. Ann Oncol.

[15] Lee DW, Kim KJ, Han SW, Lee HJ, Rhee YY, Bae JM (2015). KRAS Mutation is Associated with Worse Prognosis in Stage III or High-risk Stage II Colon Cancer Patients Treated with Adjuvant FOLFOX. Ann Surg Oncol.

[16] Ogino S, Meyerhardt JA, Irahara N, Niedzwiecki D, Hollis D, Saltz LB (2009). KRAS mutation in stage III colon cancer and clinical outcome following intergroup trial CALGB 89803. Clin Cancer Res.

[17] Hutchins G, Southward K, Handley K, Magill L, Beaumont C, Stahlschmidt J (2011). Value of mismatch repair, KRAS, and BRAF mutations in predicting recurrence and benefits from chemotherapy in colorectal cancer. J Clin Oncol.

[18] Andreyev HJ, Norman AR, Cunningham D, Oates JR, Clarke PA (1998). Kirsten ras mutations in patients with colorectal cancer: the multicenter “RASCAL” study. J Natl Cancer Inst.

[19] Andreyev HJ, Norman AR, Cunningham D, Oates J, Dix BR, Iacopetta BJ (2001). Kirsten ras mutations in patients with colorectal cancer: the ‘RASCAL II’ study. Br J Cancer.

[20] Inoue Y, Saigusa S, Iwata T, Okugawa Y, Toiyama Y, Tanaka K (2012). The prognostic value of KRAS mutations in patients with colorectal cancer. Oncol Rep.

[21] Nash GM, Gimbel M, Cohen AM, Zeng ZS, Ndubuisi MI, Nathanson DR (2010). KRAS mutation and microsatellite instability: two genetic markers of early tumor development that influence the prognosis of colorectal cancer. Ann Surg Oncol.

[22] Phipps AI, Buchanan DD, Makar KW, Win AK, Baron JA, Lindor NM (2013). KRAS-mutation status in relation to colorectal cancer survival: the joint impact of correlated tumour markers. Br J Cancer.

[23] Perez-Ruiz E, Rueda A, Pereda T, Alcaide J, Bautista D, Rivas-Ruiz F (2012). Involvement of K-RAS mutations and amino acid substitutions in the survival of metastatic colorectal cancer patients. Tumour Biol.

[24] Tie J, Lipton L, Desai J, Gibbs P, Jorissen RN, Christie M (2011). KRAS mutation is associated with lung metastasis in patients with curatively resected colorectal cancer. Clin Cancer Res.

